# Multisystem inflammatory syndrome associated with SARS-CoV-2 infection in 45 children: a first report from Iran

**DOI:** 10.1017/S095026882000196X

**Published:** 2020-08-28

**Authors:** Setareh Mamishi, Zahra Movahedi, Mohsen Mohammadi, Vahid Ziaee, Mahmoud Khodabandeh, Mohammad Reza Abdolsalehi, Amene Navaeian, Hosein Heydari, Shima Mahmoudi, Babak Pourakbari

**Affiliations:** 1 Department of Infectious Diseases, Pediatrics Center of Excellence, Children's Medical Center, Tehran University of Medical Sciences, Tehran, Iran; 2 Pediatric Infectious Disease Research Center, Tehran University of Medical Sciences, Tehran, Iran; 3 Department of Pediatric Infectious Disease, Faculty of Medicine, Qom University of Medical Sciences and Health Services, Qom, Iran; 4 Non-Communicable Pediatric Diseases Research Center, Health Research Institute, Babol University of Medical Sciences, Babol, Iran; 5 Pediatric Rheumatology Research Group, Rheumatology Research Center, Tehran University of Medical Sciences, Tehran, Iran; 6 Department of Pediatrics, Tehran University of Medical Sciences, Tehran, Iran

**Keywords:** Children, COVID-19, Multisystem Inflammatory Syndrome

## Abstract

During the coronavirus disease 2019 (COVID-19) pandemic, a new phenomenon manifesting as a multisystem inflammatory syndrome in children (MIS-C) which has a similar clinical presentation to Kawasaki disease, toxic shock syndrome and severe sepsis has emerged. Although the number of MIS-C reports is increasing, rare reports in Asia is still available. To our knowledge, this study is the largest series of published MIS-C cases in Iran. We performed a retrospective study of all patients with case definition for MIS-C admitted to the three paediatric hospitals in Iran. All of these hospitals are located within the most active COVID-19 pandemic areas (Tehran, Qom and Mazandaran) in Iran. Demographic characteristics, clinical data, laboratory findings, imaging and echocardiographic findings, treatment and outcomes were collected. Between 7 March and 23 June 2020, 45 children were included in the study. The median age of children was 7 years (range between 10 months and 17 years). Common presenting symptoms include fever (91%), abdominal pain (58%), nausea/vomiting (51%), mucocutaneous rash (53%), conjunctivitis (51%) and hands and feet oedema (40%) with median duration of symptoms prior to presentation of 5 (interquartile range (IQR) 3, 7) days. Fifty-three percent of children showed lymphopaenia. Overall, the majority of cases at admission had markedly elevated inflammatory markers erythrocyte sedimentation rate (ESR) (95.5%) and C-reactive protein (CRP) (97%). Ferritin was abnormal in 11 out of 14 tested patients (73%), and it was highly elevated (>500 ng/ml) in 47% of cases. Median fibrinogen level was 210 (IQR 165, 291) mg/dl, D-dimer was 3909 (IQR 848, 4528) ng/ml and troponin was 0.6 (IQR 0.1, 26) ng/ml, respectively. Twenty out of 31 patients (64.5%) had hypoalbuminaemia. In addition, hyponatraemia was found in 64% of cases. Twenty-five patients (56%) presented with cardiac involvement and acute renal failure was observed in 13 cases (29%). Pleural, ascitic, ileitis and pericardial effusions were found in 18%, 11%, 4% and 2% of cases, respectively. In conclusion, this is a first large case series of hospitalised children who met criteria for MIS-C in Iran. There was a wide spectrum of presenting signs and symptoms; evidence of inflammation with abnormal values of CRP, ESR, D-dimer, ferritin and albumin; and multi-organ involvement.

## Introduction

The coronavirus disease 2019 (COVID-19) pandemic was first reported in China and then spread throughout the world. At first, paediatricians though that children might be only mildly symptomatic, while during the pandemic, wide spectrums of presenting signs and symptoms as well as atypical findings are reported [1]. Recently, a new phenomenon manifesting as a multisystem inflammatory syndrome in children (MIS-C) which has a similar clinical presentation to Kawasaki disease, toxic shock syndrome and severe sepsis has emerged [2, 3].

MIS-C associated with severe acute respiratory syndrome coronavirus 2 (SARS-CoV-2) have some similar clinical features to Kawasaki, including fever, dilation of conjunctival blood vessels, rash and redness of the oropharynx [4]. However, MIS-C affects older children and adolescents. Moreover, some laboratory findings including leucopaenia is not found usually in Kawasaki disease [4].

Children with MIS-C more often have positive test for antibody to SARS-CoV-2 than for virus using nasopharyngeal real-time reverse transcription polymerase chain reaction (rRT-PCR) assay [5, 6].

Although the pathogenesis of SARS-CoV-2 is not yet fully understood, immune misdirection may lead to higher replication of virus and tissue damages [7]. MIS-C is mainly related to a hyperinflammatory responses triggered by SARS-CoV-2. Cytokine storm experienced by children with MIS-C may derive from the ability of SARS-CoV-2 to block types I and III interferon responses as well as strong expression of cytokines and chemokines [8, 9].

According to the French surveillance study, MIS-C might occur in fewer than two per 10 000 children [10]. Although case series of MIS-C have now been reported from the United States [5, 11, 12] and Europe including the UK [2, 13], Spain [14], Italy [6], France [10, 15–17] and Switzerland [18], only a few cases have been reported from Asia [19, 20]. The aim of this study was to describe the clinical and laboratory characteristics of 45 patients who met criteria for MIS-C in Iran.

## Materials and methods

This study was approved by the Ethics Committee of Tehran University of Medical Sciences, Tehran, Iran (IR.TUMS.VCR.REC.1399.057) and signed informed consent was obtained from all patients or from their parents/legal guardians who participated in the study.

We performed a retrospective study of all patients with case definition for MIS-C was according to the Centers for Disease Control and Prevention (CDC) [21] admitted to the three children's hospitals in Iran. All of these hospitals are located within the most active COVID-19 pandemic areas (Tehran, Qom and Mazandaran) in Iran. Tehran is the most populous city in Iran and Western Asia. Children's Medical Center, Tehran, Iran is one of the most experienced sub-specialised hospitals in the country that offers high quality and specialised therapeutic services to neonates, infants and children throughout country. Qom, the seventh largest city in Iran, is located on the south of Tehran and the first confirmed cases of SARS-CoV-2 infections were reported there.

Babol, one of the most important cities in the north of Iran, is the capital of Mazandaran Province and located on the north-east of Tehran. All of the three children's hospitals have the daily incidence of 3 to 5 suspected/confirmed hospitalised COVID-19 cases.

According to CDC case definition, a case with MIS-C was defined as:

- An individual aged <21 years presenting with fever, laboratory evidence of inflammation including an elevated level of C-reactive protein (CRP), erythrocyte sedimentation rate (ESR), fibrinogen, procalcitonin, D-dimer, ferritin, lactic acid dehydrogenase (LDH) or interleukin 6 (IL-6), elevated neutrophils, reduced lymphocytes and low albumin, and evidence of clinically severe illness requiring hospitalisation, with more than two multisystem organ involvement (cardiac, renal, respiratory, haematologic, gastrointestinal, dermatologic or neurological);
- No alternative plausible diagnoses and
- Positive for current or recent SARS-CoV-2 infection by RT-PCR or serology test.

A confirmed case of COVID-19 was defined as a positive result of SARS-CoV-2 rRT-PCR or positive SARS-CoV-2 antibody assay. SARS-CoV-2 rRT-PCR testing using a nasopharyngeal swab was performed for all patients and in suspected cases with negative SARS-CoV-2 rRT-PCR, SARS-CoV-2 antibody assay were performed.

The RNA of the collected samples on the swab was then extracted using a SinaPure™ Viral kit (Sinaclon, Iran) and cDNA template synthesis was performed using PrimeScript™ RT reagent Kit (TaKaRa, Japan). The rRT-PCR was performed according to the CDC protocol using the same primers and probes as in the CDC 2019-Novel Coronavirus (2019-nCoV) rRT-PCR Diagnostic Panel [22]. These included N1 and N2 probes that were selected from the regions of the virus nucleocapsid gene and RNase P (RP) was used as an internal control.

The rRT-PCR assay was performed using the Premix Ex Taq™ (Probe qPCR, TaKaRa, Japan) following the manufacturer's instructions. Each sample was run duplicated with positive and negative controls. The PCR cycle was run as follows: 95 °C for 3 min, followed by 45 cycles of 95 °C for 3 s, and 58 °C for 30 s. A cycle threshold value (Ct value) of less than 37 was defined as a positive test result.

Detection of SARS-CoV-2 antibodies was performed using SARS-CoV-2 immunoglobulin M (IgM) ELISA kits (Pishtaz Teb, Iran, http://pishtazteb.com) and SARS-CoV-2 IgG ELISA kits (Pishtaz Teb, Iran http://pishtazteb.com) according to the manufacturer's protocol.

Patients with MIS-C were divided into three groups including Kawasaki-like, toxic shock-like and sepsis-like disease. Kawasaki-like disease was defined as the presence of fever for ≥3 days but ≤10 days and who fulfilled ≥4 of 5 diagnostic criteria (rash, conjunctival injection, cervical lymphadenopathy, changes in the oral mucosa and changes in the extremities) or three criteria plus coronary artery abnormalities documented through echocardiography. Toxic shock-like disease was defined as the subset with cardiovascular dysfunction, which included basal systolic blood pressure of at least 20%, or the appearance of signs of peripheral hypoperfusion [23, 24] and sepsis-like was defined as life-threatening organ dysfunction caused by a dysregulated host response to infection [25].

Demographic characteristics, clinical data (comorbidities, delay between symptom onset and hospital admission, baseline symptoms and physical signs), laboratory findings (including leucocyte, neutrophil and lymphocyte counts, CRP, troponin levels, ESR, D-dimer, blood urea nitrogen (BUN), serum creatinine, liver enzymes, fibrinogen, ferritin, LDH, creatine phosphokinase (CPK), sodium, potassium, albumin), imaging and echocardiographic findings, treatment and outcomes were collected.

### Statistical analysis

All statistical analyses were performed using SPSS (Statistical Package for the Social Sciences) version 13.0 software (SPSS Inc.). Categorical variables were described as frequency rates and percentages, and continuous variables were described using median and interquartile range (IQR) values.

## Results

Between 7 March and 23 June 2020, 45 children (25 cases from Tehran, 13 cases from Qom and 7 cases from Mazandaran) who had been admitted to three children's hospitals in Iran and met criteria for MIS-C were included in the study.

Demographics and baseline clinical characteristics of the patients are presented in Table 1. The median age of the children was 7 years (range between 10 months and 17 years) and 53% of them were male. Comorbid conditions including acute lymphocytic leucaemia, chronic kidney disease, underlying seizure disorder, cerebral palsy, cardiovascular disease and Budd–Chiari syndrome were present in six (13%) patients. Common presenting symptoms include fever (91%), abdominal pain (58%), nausea/vomiting (51%), mucocutaneous rash (53%), conjunctivitis (51%) and hands and feet oedema (40%) with the median duration of symptoms prior to presentation of 5 (IQR 3, 7) days.

**Table 1.** Demographics, clinical findings, imaging and echocardiogram findings, treatment and outcome of patients with MIS-C

Parameter	Value
Age in years, median (IQR)	7 (4–9.9)
Male, no. (%)	24 (53)
Comorbid conditions, no. (%)	6 (13)
Hospital stay, median (IQR)	8 (6–11)
Symptoms	
Duration of symptoms in days prior to admission	5 (3–7)
Fever, no. (%)	41 (91)
Cough, no. (%)	16 (36)
Mucocutaneous rash, no. (%)	24 (53)
Conjunctivitis, no. (%)	23 (51)
Nausea/vomiting, no. (%)	23 (51)
Abdominal pain, no. (%)	26 (58)
Myalgy, no. (%)	17 (38)
Tachypnoea, no. (%)	8 (18)
Cervical lymphadenopathy, no. (%)	9 (20)
Tiredness, no. (%)	11 (24)
Diarrhoea, no. (%)	16 (36)
Sore throat, no. (%)	7 (16)
Hands and feet oedema, no. (%)	18 (40)
Shortness of breath, no. (%)	10 (22)
Known COVID+ contact, no. (%)	14 (31)
Imaging and echocardiogram results	
Pleural effusions, no. (%)	8 (18)
Ascites, no. (%)	5 (11)
Ileitis, no. (%)	2 (4)
Cardiomegaly, no. (%)	2 (4)
Coronary dilation, no. (%)	14 (31)
Pericardial effusion, no. (%)	1 (2)
Myocarditis, no. (%)	8 (18)
Treatment	
IVIG, no. (%)	18 (48)
Steroids, no. (%)	27 (60)
Mortality, no. (%)	5 (11)

A majority of patients with MIS-C showed Kawasaki-like disease (*n* = 31, 69%), while toxic shock-like and sepsis-like diseases were observed in 11% (*n* = 5) and 20% of the cases (*n* = 9). Abdominal pain was observed more in case with Kawasaki-like disease and sepsis-like disease compared to the toxic shock-like disease (Table 2).

**Table 2.** Clinical findings of 45 cases with MIS-C according to the different groups (Kawasaki-like, Toxic shock-like, Sepsis-like)

Symptoms	Kawasaki-like (*N* = 31)	Toxic shock-like (*N* = 5)	Sepsis-like (*N* = 9)
Fever, no. (%)	29 (94)	5 (100)	7 (78)
Cough, no. (%)	12 (39)	2 (40)	2 (22)
Mucocutaneous rash, no. (%)	18 (58)	3 (60)	3 (33)
Conjunctivitis, no. (%)	20 (65)	1 (20)	2 (22)
Nausea/vomiting, no. (%)	16 (52)	3 (60)	4 (44)
Abdominal pain, no. (%)	19 (61)	1 (20)	6 (67)
Myalgy, no. (%)	14 (45)	2 (40)	1 (11)
Tachypnoea, no. (%)	7 (23)	0	1 (11)
Cervical lymphadenopathy, no. (%)	9 (295)	0	0
Tiredness, no. (%)	6 (19)	2 (40)	3 (33)
Diarrhoea, no. (%)	11 (35)	2 (40)	3 (33)
Sore throat, no. (%)	6 (19)	1 (20)	0
Hands and feet oedema, no. (%)	14 (45)	1 (20)	3 (33)
Shortness of breath, no. (%)	6 (19)	0	4 (44)

Fourteen patients (31%) had close contact with a family member with proven COVID-19. Results from SARS-CoV-2 rRT-PCR tests were positive in 22% (*n* = 10) (Table 2) and SARS-CoV-2 antibody assay was positive in 35 of 45 patients (78%).

Laboratory test results of on admission are presented in Table 3. Twenty-nine children had normal white blood cell (WBC) counts and thrombocytopaenia was found in 17 patients (39%). Elevated levels of CPK, LDH, BUN and transaminases were found in 10%, 11%, 29% and 56% of patients, respectively.

**Table 3.** Laboratory findings of patients with MIS-C

Parameter	Value
SARS-CoV-2 PCR positive	10 (22)
SARS-CoV-2 antibody positive	35 (78)
WBCs in cells/μl, median (IQR)	8300 (6425– 12 100)
Haemoglobin in g/dl, median (IQR)	11.1 (9.8–12.3)
Platelets in thousands/μl, median (IQR)	167 000 (89 000– 275 000)
Absolute lymphocyte count in thousands/μl, median (IQR)	1260 (655–2700)
Absolute neutrophil count in thousands/μl, median (IQR)	6600 (4400–9050)
ESR in mm/h, median (IQR)	35.5 (22.5–54.5)
CRP in mg/l, median (IQR)	67 (29.5–102)
BUN in mg/dl, median (IQR)	15 (10–22.5)
Serum creatinine in mg/dl, median (IQR)	0.6 (0.5–0.8)
Aspartate aminotransferase in U/l, median (IQR)	37 (20–62.5)
Alanine aminotransferase in U/l, median (IQR)	32 (25–61.5)
Fibrinogen in mg/dl, median (IQR)	210 (165–291)
Ferritin in ng/ml, median (IQR)	453 (179–1450)
Troponin in ng/ml, median (IQR)	0.6 (0.1–26)
D-Dimer in ng/ml, median (IQR)	3909 (848–4528)
Lactate dehydrogenase in IU/l, median (IQR)	535 (450–697)
CPK in U/l, median (IQR)	51 (26–95.5)
Sodium in meq/l, median (IQR)	132.5 (129–135)
Potassium in meq/l, median (IQR)	3.8 (3.6–4.1)
Albumin in g/dl, median (IQR)	3.4 (3.0–4.2)

IQR, interquartile range.

Median WBC count was 8300 (IQR 6425, 12 100) cells/μl. Lymphopaenia was found in 24 patients (53%). Markers of inflammation were elevated with median CRP 67 (IQR 29.5, 102) mg/l, ESR 35.5 (IQR 22.5, 54.5) mm/h and ferritin 453 (IQR 179, 1450) ng/ml. Median fibrinogen was 210 (IQR 165, 291) mg/dl, D-dimer was 3909 (IQR 848, 4528) ng/ml and troponin was 0.6 (IQR 0.1, 26) ng/ml. Ferritin was abnormal in 11 of 14 tested patients (73%), and highly elevated (>500 ng/ml) in 47% of cases.

Overall, the majority of cases at admission had markedly elevated inflammatory markers ESR (95.5%) and CRP which is considered as a surrogate marker of IL-6 (97%). Twenty out of 31 patients (64.5%) had hypoalbuminaemia and hyponatraemia was found in 64% of cases.

Imaging and echocardiogram findings are presented in Table 1. Twenty-five patients (56%) presented with cardiac involvement. Coronary artery dilations and myocarditis were detected in 31% and 18% of the patients, respectively. Acute renal failure was observed in 13 cases (29%).

Twenty-seven patients (60%) received steroid treatment with methylprednisone (dose range, 2–30 mg/kg per day) and 18 patients (48%) received intravenous immunoglobulins (IVIG) (dose range, 2–4 g/kg). The mortality of 11% was reported (five cases); four of them had underlying diseases (acute lymphocytic leucaemia, chronic kidney disease, cerebral palsy and Budd–Chiari syndrome), while no comorbid conditions were found in one case. Among these five cases, two showed sepsis-like disease and toxic shock-like disease were found in three cases.

## Discussion

However, since late April 2020, there have been an increasing number of reports on children with MIS-C, a few reports in Asia is still available. To our knowledge, this study is, to date, the largest series of published MIS-C cases in Iran.

The median age of children was 7 years (range between 10 months and 17 years). Similar to previous reports, patients with MIS-C tend to be older than 5 years old [3, 5, 6, 10, 17, 26] and it is in contrast to the epidemiology of Kawasaki disease that approximately 80% of cases occurring in children <5 years of age [4]. Median time from the onset of symptoms prior to hospitalisation was 5 days that was similar to previous reports [15, 26].

Twenty-two percent of our cases had positive SARS-CoV-2 rRT-PCR result. In Kaushik *et al*. study, 33% tested positive on SARS-CoV-2 rRT-PCR [5], and in Verdoni *et al*. [6] and Whittaker *et al*. study [13], it was positive in 20% and 26% of patients, respectively; therefore, antibody testing might be considered as a main tool in determining the relationship of COVID-19 to MIS-C [3].

Although no fatalities [12, 15, 17] or relatively few reported cases of paediatric deaths attributed to MIS-C [5, 13] was reported, in this study 11% of patients died.

Gastrointestinal signs and symptoms appear predominantly as presenting features of MIS-C [12, 15, 17, 26].

Mucocutaneous rash (53%), conjunctivitis (51%) and hands and feet oedema (40%) were the other prevalent findings that were similar to the report of Whittaker *et al*. in the UK [13].

Our study showed pleural, ascitic, ileitis and pericardial effusions in 18%, 11%, 4% and 2% of cases, respectively; which indicate a diffuse inflammatory process of MIS-C [2].

In our study, 54% of children showed lymphopaenia. In Cheung *et al*.'s study, 71% of cases showed lymphopaenia [26]. The majority of cases at admission had markedly elevated inflammatory markers including ESR and CRP and mildly decreased albumin that was similar to previous reports [2, 3, 5, 12, 13, 15, 26, 27]. Hypoalbuminaemia was found in 64.5% of patients that was higher than previous report [15]. Liver enzymes were abnormal in five cases (31%). Ferritin was abnormal in 73% of patients, and highly elevated (>500 μg/l) in 47% of cases that was in consistent with the previous report [11, 15]. Hyponatraemia was found in 64% of the cases that was similar to Chiotos *et al*.'s study in the UK [11]. Although high level of abnormal D-dimers have been associated with higher thrombosis rates mainly in adults with COVID-19 [3], in our study high level of D-dimers was observed in cases MIS-C.

MIS-C is mainly as a result of an antibody-mediated of immune system and cytokine storm leading to cardiac or renal failures [5, 13, 15, 28]. The increased level of enzymes in the liver, heart and kidneys might lead to the occurrence of multi-organ failure in MIS-C [7]. In our study, 56% of patients presented with cardiac involvement that was similar to the reports of Verdoni *et al*.'s study [6]. In addition, 29% of patients presented with dilated coronary arteries that was consistent with previous reports [15, 17]. Acute renal failure was observed in 13 cases (29%), while in Grimaud *et al*. [16] and Pouletty *et al*. [15] studies, it was reported in 70% and 56% of cases, respectively.

Although there are currently no CDC recommendations regarding the treatment of MIS-C, anti-inflammatory drugs including IVIG and steroids are mainly prescribed. However, due to the high cost, insurance carriers and government agencies may not approve IVIG therapy, even for some disorders with approved indications which showed evidence-based efficacy [29]. Glucocorticoid are widely used for treatment of patients with SARS [7] and COVID-19 [30] for controlling of cytokine production, inflammatory response and accumulation of cells and fluids.

In our study, 60% and 48% of MIS-C cases have been treated with steroids and IVIG, respectively. It has been reported that the administration of corticosteroids might decrease mortality in severe pneumonia [31]. In our study, no death was observed in cases that were treated with corticosteroids. Overall, equal or even better efficacy (defined as improvement or absence of worsening the condition after starting of treatment) and the lower price of steroids compare to IVIG, make steroids as better choice for treatment of MIS-C.

In conclusion, in this first large case series of hospitalised children who met criteria for MIS-C in Iran, there was a wide spectrum of presenting signs and symptoms; evidence of inflammation with abnormal values of CRP, ESR, D-dimer, ferritin and albumin; and multi-organ involvement.

## Data Availability

Requests for access to the data that support this study should be made to the corresponding author, SM.
